# Genetics of *ABCB1* in Cancer

**DOI:** 10.3390/cancers15174236

**Published:** 2023-08-24

**Authors:** Katie T. Skinner, Antara M. Palkar, Andrew L. Hong

**Affiliations:** 1 Department of Pediatrics, Emory University School of Medicine, Atlanta, GA 30322, USA; katie.skinner@emory.edu (K.T.S.); antara.malhar.palkar@emory.edu (A.M.P.); 2 Aflac Cancer and Blood Disorders Center, Children’s Healthcare of Atlanta, Atlanta, GA 30322, USA; 3 Winship Cancer Institute, Emory University School of Medicine, Atlanta, GA 30322, USA

**Keywords:** ABCB1, multidrug resistance, chemotherapy, genetics, epigenetics

## Abstract

**Simple Summary:**

Overexpression of *ABCB1* has been identified in a wide range of multidrug-resistant cancers. *ABCB1* can become upregulated in many ways, and understanding these mechanisms of upregulation could provide novel insights into cancer multidrug resistance. In this review, we summarize genetic and epigenetic mechanisms of *ABCB1* upregulation in cancer and highlight areas that may be relevant for future research.

**Abstract:**

*ABCB1*, also known as *MDR1*, is a gene that encodes P-glycoprotein (P-gp), a membrane-associated ATP-dependent transporter. P-gp is widely expressed in many healthy tissues—in the gastrointestinal tract, liver, kidney, and at the blood–brain barrier. P-gp works to pump xenobiotics such as toxins and drugs out of cells. P-gp is also commonly upregulated across multiple cancer types such as ovarian, breast, and lung. Overexpression of *ABCB1* has been linked to the development of chemotherapy resistance across these cancers. In vitro work across a wide range of drug-sensitive and -resistant cancer cell lines has shown that upon treatment with chemotherapeutic agents such as doxorubicin, cisplatin, and paclitaxel, *ABCB1* is upregulated. This upregulation is caused in part by a variety of genetic and epigenetic mechanisms. This includes single-nucleotide variants that lead to enhanced P-gp ATPase activity without increasing *ABCB1* RNA and protein levels. In this review, we summarize current knowledge of genetic and epigenetic mechanisms leading to *ABCB1* upregulation and P-gp-enhanced ATPase activity in the setting of chemotherapy resistance across a variety of cancers.

## 1. Introduction

The ATP-binding cassette (ABC) family of transporters uses energy in the form of ATP to transport substrates against a concentration gradient. One well-studied member of this family is *ABCB1*, which encodes P-glycoprotein (P-gp). The role of P-gp in healthy tissue is well established, where it has been found to be expressed on organs and tissues that have roles in the detoxification of xenobiotics and a wide variety of drugs, including anti-cancer drugs [1]. Many chemotherapeutic and targeted agents, such as daunorubicin, docetaxel, doxorubicin, etoposide, imatinib, mitoxantrone, paclitaxel, sunitinib, teniposide, topotecan, vinblastine, and vincristine [2,3] have been established as P-gp substrates, and overexpression of this membrane-bound efflux pump prevents accumulation of these drugs within cells, mediating the development of resistance. Inappropriate upregulation of P-gp in cancer cells leads to the development of therapy-resistant cancers.

In this review, we summarize current knowledge on P-gp structure, function, localization, and substrate specificity. We also focus on the genetics of *ABCB1*, reviewing publicly available data to understand the prevalence of *ABCB1* alterations across different cancers, alongside common mechanisms by which *ABCB1* has been found to be upregulated in multidrug-resistant cancers.

## 2. Overview of ABC Family of Transporters

*ABCB1* is a member of the ATP-binding cassette (ABC) family of transporter proteins [4]. P-gp, encoded by *ABCB1*, was first identified in 1976 by Juliano and Ling [5]. In this study, researchers previously isolated Chinese hamster ovarian tissue cells that demonstrated resistance to colchicine, an anti-inflammatory drug. They also observed that these cells were resistant to other non-related compounds such as vinblastine and puromycin [5]. Surface labeling studies revealed a 170 kDa component on the cell surface that was not present on non-resistant wild-type cells [5]. Upon metabolic labeling studies, the component was identified as a glycoprotein [5]. As these mutant cells displayed a decreased permeability, this component was henceforth named “permeability glycoprotein” or P-glycoprotein (P-gp); however, later studies indicated that P-gp functions as an efflux pump, rather than altering permeability status in cells [6,7,8]. In 1985, the *ABCB1* gene was cloned, and, using this cDNA as a probe, researchers were able to show that *ABCB1* DNA was amplified in the vinblastine-resistant leukemia cell line CEM/VLB compared to its parental counterpart, CCRF-CEM [9].

**Figure 1:**
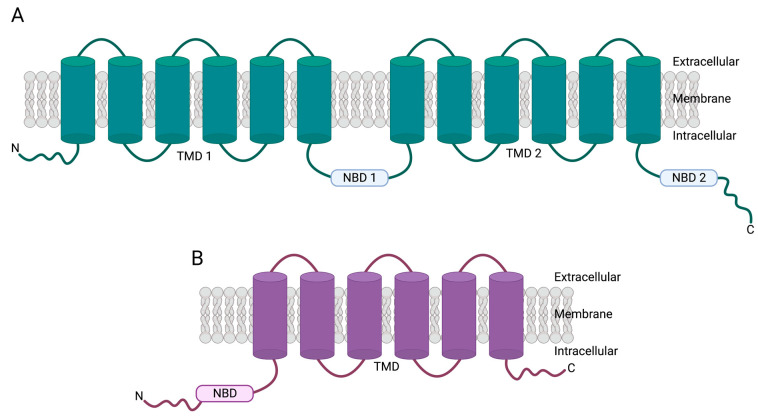
Example protein structures: (**A**) full transporter, P-glycoprotein (P-gp/*ABCB1*); and (**B**) half transporter breast-cancer-resistance protein (BCRP/*ABCG2*). NBD: nucleotide-binding domain; TMD: transmembrane domain. Created with BioRender.com (accessed on 7 August 2023).

More broadly, this family of transporters consists of seven sub-families: ABCA to ABCG [4,10]. Within these seven sub-families, there are, to date, forty-eight transporters [4,11]. An additional gene in the ABC family, ABCC13, is expected to produce a nonfunctional protein [12]. One way ABC transporters can be grouped is by structure, whereby they exist as either full or half transporters [13]. Generally, full transporters consist of two transmembrane domains (TMDs) and two nucleotide-binding domains (NBDs) [13] (Figure 1A). On the other hand, half transporters consist of one TMD and one NBD, meaning to function correctly, half transporters are required to form homodimers or heterodimers [14,15,16,17] (Figure 1B).

## 3. *ABCB1* Genetics

**Figure 2:**
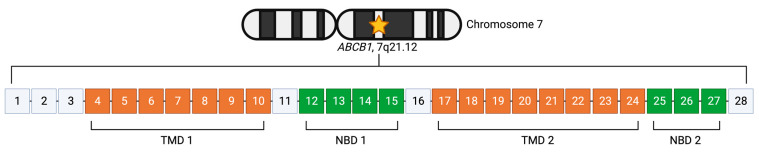
Location and structure of the *ABCB1* gene. Gene structure of *ABCB1* as in the NCBI entry NM_001348946.2 and Ensembl entry ENST00000622132.5. This is the only gene variant that is common to both databases. Orange depicts exons that code for transmembrane domains (TMDs) and green depicts exons that code for nucleotide-binding domains (NBDs). Created with BioRender.com (accessed on 13 July 2023).

The *ABCB1* gene is located on chromosome 7q21.12 [NCBI, Gene ID: 5243, GRCh38.p13; Ensembl, Gene ID: ENSG00000085563, GRCh38.p13] and has been found to have two promoters—a proximal “downstream” promoter, and a distal “upstream” promoter [18]. This was first characterized by Chen et al., with the proximal promoter being located in the first exon, and the distal promoter in the second exon [18]. Transcripts derived from the upstream promoter will have 29 exons, and those derived from the downstream promoter will have 28 exons [18] (Figure 2). The literature suggests that the 28 exon *ABCB1* is most commonly produced by normally functioning cells, whereas drug-resistant cancer cells might more frequently produce the slightly longer 29 exon form [19,20,21,22]. This is discussed in more depth in Section 5.7. Between NCBI and Ensembl, the 28 exon, 1280 amino acid protein (Table 1, NM_001348946.2 and ENST00000622132.5) is the only isoform to be shared between the two databases.

Besides the one shared database entry (Table 1, NM_001348946.2 and ENST00000622132.5), there are discrepancies regarding mRNA and protein isoforms listed between the NCBI and Ensembl databases. For example, NCBI lists two isoforms containing 30 and 32 exons (Table 1, NM_001348944.2 and NM_001348945.2, respectively) that are not found in Ensembl. These contain additional exons upstream of the *ABCB1* promoters identified by Chen et al. These two sequences (Table 1, NM_001348944.2 and NM_001348945.2, respectively) were derived from bacterial artificial chromosomes, and, other than their listing in NCBI, there is no additional evidence in the literature supporting the existence of either variant; furthermore, Pappas et al. identified three hypothetical exons at the 3′ end of the *ABCB1* gene. They used RT-PCR to determine whether these hypothetical exons existed, and found no evidence that they did [23]. Taking these data into consideration, additional research is needed to support the NM_001348944.2 and NM_001348945.2 isoforms listed on NCBI. Similarly, in Ensembl, there is a truncated ABCB1 mRNA/protein listed that is not found in NCBI (Table 1, ENST00000416177.1). There is also no evidence of this isoform in the literature, and thus more research should be conducted to verify its validity.

## 4. *ABCB1* in Normal Tissues

### 4.1. Tissue Localization, Subcellular Localization, and Substrates

Given the role of P-gp as an efflux pump, it is therefore unsurprising that *ABCB1* is expressed on tissues with detoxification and/or secretory roles (Table 2). Early studies utilizing slot blot analysis of normal human tissues found that *ABCB1* was expressed on the adrenal gland, kidneys, colon, rectum, lungs, and liver [24]; other tissues, such as the brain, prostate, skin, heart, esophagus, and stomach were all found to have lower *ABCB1* expression [24]. Characterization of P-gp tissue localization at the protein level via immunohistochemistry confirmed these findings [25]. Of all the cell types where P-gp was found to be expressed, expression typically occurred on the apical surface, facing towards the excretory compartments of each of these organs [25], which is consistent with P-gp function as an efflux pump. P-gp has a long list of substrates that are still being established (summarized in Table 2). At organs such as the kidneys, liver, and colon, P-gp will pump xenobiotics, such as pesticides, and drugs such as antibiotics, calcium channel blockers, and protease inhibitors into compartments such as the proximal tubule [26,27], bile ducts [26,28], and intestinal lumen [29,30] to be removed. This function promotes the elimination of these substances from the blood and prevents them from circulating in the body, potentially causing damage. P-gp has also been found to transport hormones and steroids (Table 2), which is consistent with its high expression on the adrenal gland [24,25]. It is thought that P-gp contributes to the regulation of hormone secretion, thus modulating hormone signaling [31,32,33].

Although earlier studies did not find *ABCB1* expression within the central nervous system (regions such as the cerebral cortex, cerebellum, and spinal cord), it was later discovered that P-gp was indeed expressed on endothelial cells located at the blood–brain barrier [34]. Here, it plays an important role in protecting the brain against xenobiotics and drugs [58], such as those outlined in Table 2. Along with protecting the brain from these compounds, P-gp also has roles in the development of neurological disorders. For example, amyloid-β, the protein thought to be causative of Alzheimer’s disease, has been found to be a substrate of P-gp in humans [56], and reduced *ABCB1* expression in mice led to an accumulation of amyloid-β in the brains of these animals [59,60,61].

Lastly, research also indicates that *ABCB1* expression is prevalent in the placenta and has important roles in protecting the fetus from compounds that may impact development, such as HIV protease inhibitors [62,63], chemotherapeutic agents [63], stress hormones, and steroids [55,62]. For example, human placental tissue obtained between 6 and 10 weeks gestation had significantly higher *ABCB1* mRNA expression compared to tissue obtained between 38 and 41 weeks gestation [35]. This is consistent with a protective role for P-gp, as the fetus needs to be protected from harmful compounds during the early stages of development when it is most vulnerable.

In terms of subcellular localization, P-gp is most commonly found on the cell surface, more specifically within the plasma membrane [36,37,64]. This is consistent with its function as an efflux pump. Moreover, a few initial studies suggest that P-gp could localize intracellularly. One study found that in blood–brain endothelial cells, P-gp was expressed on the nuclear envelope in both human and rat brains [64]. Here, the authors proposed that P-gp functions to protect the nucleus and prevent DNA-drug interactions that might cause damage [64]; similarly, it has been proposed that P-gp could be expressed in the mitochondria of three different doxorubicin-resistant cancer cells lines: K562 (leukemia), PLC/PRF/5 (hepatocellular carcinoma), and MCF7 (breast). In K562 cells, researchers found that P-gp may transport substrates into the mitochondria, and they proposed that this organelle acted as a drug sequestration compartment that protected the nucleus and DNA from these toxins [65]. In PLC/PRF/5 and MCF7, however, it was found that P-gp transported substrates from the mitochondria into the cytoplasm, which is more consistent with the protective role as previously described in this review [57,66], although these studies have not been further investigated in recent years and as such, additional research is needed.

### 4.2. P-gp Mechanism of Action

It has been long established that P-gp is able to utilize ATP to transport substrates against a concentration gradient [67,68,69,70]. The exact mechanism by which P-gp transports substrates, however, is still under debate. Possible mechanisms for P-gp pumping include (1) direct extraction of substrates from the cytoplasm, (2) functioning as a flippase to remove substrates from the inner leaflet of the plasma membrane, or (3) extracting substrates from the outer leaflet to prevent them from reaching the cytoplasm.

Direct extraction proposes that P-gp functions similarly to ion-transporting ATPases, hydrolyzing ATP to maintain ion gradients across cell membranes, and extracting substrates directly from the cytoplasm [71]. In the flippase model, P-gp moves its substrates from the inner to the outer leaflet of the cell membrane [71,72,73]. In this mechanism, the substrates need to be specifically localized within each leaflet of the membrane. Once in the outer leaflet, substrates could passively diffuse into the extracellular aqueous phase or spontaneously move back to the inner leaflet. This flippase activity also requires ATP hydrolysis and is inhibited by certain substances that also inhibit drug transport by P-gp, suggesting that drugs and membrane lipids likely follow the same route through the transporter [73]. The final model suggests that P-gp extracts substrates from the outer leaflet to prevent them from reaching the cytoplasm. Experiments using fluorescent indicators and photolabeling techniques have indicated that P-gp interacts with drug molecules within the membrane, leading to the concept of P-gp as eliminating potentially harmful lipophilic compounds from the membrane [74,75]. Subsequent studies have indicated that the rate of drug efflux by P-gp is linked to the membrane concentration of the drug and inversely related to its concentration in the aqueous phase [76,77]. Experiments using a deletion mutant of P-gp have demonstrated that the transporter’s transmembrane domains alone are sufficient to bind drug substrates [78].

There is still debate as to whether (1) ATP binding and subsequent NBD dimerization or (2) ATP hydrolysis is the force driving the P-gp conformation change [67,68,69,70]. Studies addressing this question have often faced criticism due to the use of physiologically irrelevant conditions. More recent studies have tried to address these issues by studying P-gp function in near-physiological conditions [79] (i.e., in a lipid bilayer and at 37 °C) as well as in living cells [80]. While a consensus remains to be reached, advances in FRET technology, such as FRET-based biosensors, will likely enable us to underpin this mechanism sooner rather than later.

## 5. *ABCB1* in Cancers

### 5.1. P-gp Substrates: Chemotherapies and Targeted Therapies

As outlined in Section 2, it has been known for a long time that P-gp overexpressing cells are resistant to chemotherapeutic agents such as vinblastine [5,9]. Early studies that sought to establish substrates of P-gp used a photoaffinity labeling technique. Here, photoactive radiolabeled analogs of colchicine, a known P-gp substrate, were co-incubated with chemotherapeutic agents, and the efficiency of photolabeling was assessed [81], with the idea being a compound was a P-gp substrate if it competitively reduced photolabeling. The results of such an experiment indicated that agents such as vincristine, vinblastine, doxorubicin, and actinomycin D were all P-gp substrates, whereas methotrexate was not [81]. In parallel, researchers examined a large number of leukemia and lymphoma cell lines that had never been exposed to chemotherapeutic agents in culture, and tested for P-gp expression via the monoclonal antibody MRK16 [82]. Utilizing the cell lines K562 (doxorubicin-sensitive) and K562/ADM (doxorubicin-resistant) as controls, they identified three cell lines (KYO-1, HEL, and CMK) to have P-gp expression [82]. These three cell lines, as well as K562/ADM, were all found to be resistant to vincristine, vindesine, vinblastine, doxorubicin, daunorubicin, mitoxantrone, etoposide, and actinomycin-D [82]. Other cell lines with no P-gp displayed resistance to some compounds but not others, indicating P-gp-independent mechanisms of resistance [82] (potentially due to the expression of other ABC transporters, such as MRP1 and BCRP, which have an affinity for some of the same substrates as P-gp, however, this requires further investigation [83]). Nowadays, many cell lines and primary tumors have been tested for both P-gp expression and resistance to these agents, whereby a positive correlation has been established (i.e., high P-gp expression is associated with more chemotherapy resistance) across many different cancer types such as breast and ovarian cancers [84], multiple myeloma [85], osteosarcoma [86], and lung cancer [87] to name a few.

As for targeted therapies, Lee et al. conducted a high-throughput screening with the goal of identifying cytotoxic substrates of P-gp. They used the HeLa-derived cell line KB-3-1, and the colchicine-resistant subline KB-8-5-11 [88]. From this screening of 10,804 compounds, they identified 90 as potential substrates [88]. To confirm these hits, they performed IC_50_ experiments using both ovarian cell lines, as well as P-gp overexpressing HEK 293T cells, in the presence and absence of the P-gp inhibitor tariquidar. From these experiments, they confirmed multiple targeted therapies as substrates including but not limited to inhibitors of the PI3K/AKT pathway (gedatolisib and GSK-690693); cell cycle checkpoint inhibitors (AT7159 and ispinesib); and a Janus kinase 2/3 inhibitor (AT9283) [88]. Using this screening method, substrates of other ABC transporters can be identified. This information will help us to understand ABC transporter redundancy and/or compensation better, as well as cancer therapy resistance.

### 5.2. Prevalence of ABCB1 Dysregulation in Cancer

**Figure 3:**
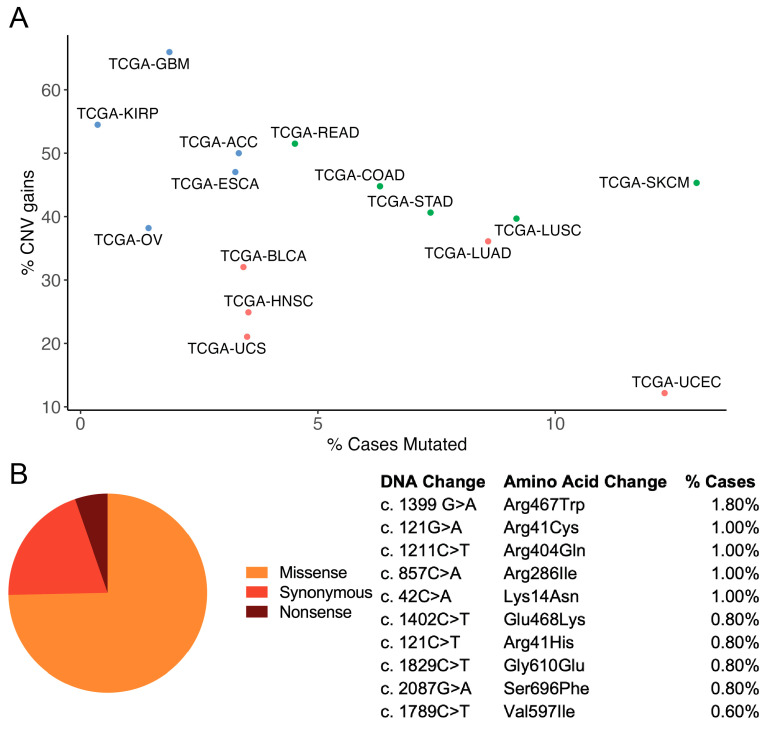
Prevalence of *ABCB1* mutations and copy number changes derived from the NCI’s publicly available GDC Data Portal: (**A**) Top 5 projects with highest prevalence of *ABCB1* mutations (single-nucleotide polymorphisms; pink points on graph), top 5 projects with highest prevalence of *ABCB1* copy number gain (blue points on graph), and top 5 projects with highest prevalence of both *ABCB1* mutations and copy number gains (green points on graph); (**B**) Proportions of missense, synonymous, and nonsense *ABCB1* mutations in cohort—table depicts top 10 missense mutations in cohort. Genomic data are based on genome assembly GRCh38p13.

To explore the prevalence of *ABCB1* dysregulation in cancer, we utilized the NCI’s publicly available GDC Data Portal [89]. Across 38 different projects, *ABCB1* mutations were found in 508 out of a total of 13,106 cases (prevalence of approximately 3.9%). Of these 508 patients, 472 had age data, whereby approximately 97% were over the age of 18, illustrating the lack of knowledge we currently have regarding *ABCB1* dysregulation in pediatric cancers. *ABCB1* mutations occurred most frequently in lung, skin, and uterine malignancies (Figure 3A). Of all 508 cases, 550 total mutations were identified. Of these mutations, almost 70% were missense (Figure 3B), with the most common missense mutation being *ABCB1* R467W (frequency of 1.8% in a cohort of patients with an *ABCB1* mutation). Interestingly, 2 of the 459 adult cases were derived from recurrent tumors, each with a different *ABCB1* missense mutation: *ABCB1* A252E (lung) and *ABCB1* A599T (brain). Although it is possible that the presence of these mutations is not significant, the functional consequences of these SNPs are unknown, and further study would be required to determine whether they play a role in the tumorigenesis and recurrence of these cancers.

As for gene level copy number gains, across 33 projects, 3345 out of 10,785 total cases had copy number gains of the *ABCB1* gene (frequency of 31%). Malignancies that saw the highest frequency of gains originated in the testis, brain, kidneys, adrenal gland, and intestine (Figure 3A). Of these cases, 34 were recurrent tumors, with 30 harboring an *ABCB1* copy number gain, and 4 harboring an *ABCB1* copy number loss. Of these 34, 30 received treatment, and of these 30, 28 had copy number gains of *ABCB1*, with 2 cases harboring *ABCB1* copy number losses. Currently, it is unclear if increases in *ABCB1* gene expression mirror this increase in gene level copy number. As a result, it remains unknown what the functional significance of these copy number changes is. Additional investigation is required to ascertain the significance of *ABCB1* gene level copy number increases and how this might relate to cancer therapy resistance.

As aforementioned, much of this data occurs in adult patients, with little to no data on pediatric cases; additionally, the role of *ABCB1* in pediatric cancer has also not yet been characterized, so future research should focus on understanding the influence of *ABCB1* mutations or copy number gains in this cohort.

### 5.3. Structural Variants Leading to ABCB1 Upregulation: Chromosome 7 Amplifications

Aneuploidies, or alterations in chromosomal copy number, are commonly found in cancer. Typically, regions containing oncogenes are found to be amplified, and regions containing tumor suppressor genes are lost. As it became more established that *ABCB1* was linked to multidrug resistance, researchers sought to understand how the gene was becoming overexpressed. A study by Wang et al. generated 11 multidrug-resistant sublines from a total of 6 ovarian cancer cell lines. These lines were resistant to either paclitaxel or docetaxel [90]. Using cDNA microarrays, they found that a cluster of genes located at chromosome 7q21.11-13 was overexpressed in nine of the resistant lines [90]. In six of these nine lines, they found evidence of copy number amplifications of this region and attributed this to the increased expression of this gene cluster, which included *ABCB1* [90]. Similarly, Yabuki et al. generated a paclitaxel-resistant subline of the non-small cell lung carcinoma cell line NCI-H460 and observed both overexpression of *ABCB1* and regional amplifications of chromosome 7p21.12, 11- to 13-fold higher than seen in the parental, non-resistant cell line. Analogous findings have also been found in the docetaxel-resistant breast cancer cell line MCF7 [21], and the doxorubicin-resistant leukemic cell line, K562 [91]. This research shows that *ABCB1* overexpression can be due to chromosome 7 amplifications across multiple different cancer types that are resistant to different chemotherapeutic agents.

### 5.4. Structural Variants Leading to ABCB1 Upregulation: Gene Fusions

**Figure 4:**
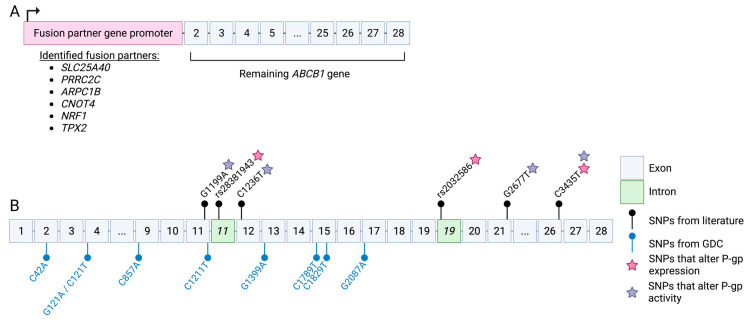
Genetics aberrations in *ABCB1*: (**A**) Partners of *ABCB1* fusions. Commonly, the promoter of a fusion partner will fuse with exon 2 onwards of *ABCB1*, promoting *ABCB1* overexpression; (**B**) Distribution of *ABCB1* single-nucleotide polymorphisms (SNPs) discussed in this review. Created with BioRender.com (accessed on 13 July 2023).

As well as chromosome amplifications, transcriptional fusions of the N-terminus of *ABCB1* to a truncated C-terminus of another gene, resulting in *ABCB1* upregulation have been identified. In 2015, Patch et al. conducted a whole-genome sequencing analysis of 92 chemoresistant ovarian cancer patient samples, with additional primary tumor tissue that was chemo-sensitive. From this analysis, they found a transcriptional fusion between *ABCB1* and the solute carrier, *SLC25A40* in two patients [92]. This fusion placed ABCB1 under the *SLC25A40* promoter, causing upregulation in *ABCB1* and downregulation in *SLC25A40*, due to deletion of the rest of the gene [92]. Researchers confirmed that this fusion was not present in the primary, chemo-sensitive, tumors, and stated that these patients had failed to respond to treatments containing P-gp substrates [92]. The same group then sought to characterize this fusion in an even larger ovarian cohort comprising patients with recurrent disease, as well as a smaller group of breast cancer patients [93]. Analysis of this cohort found additional fusions of *ABCB1* with other genes such as *PRRC2C*, *ARPC1B*, and *CNOT4* in ovarian tissue, and *NRF1* and *TPX2* in breast tissue, alongside the original *SLC25A40-ABCB1* fusion identified in the prior study [93]. Many of these novel fusions were found to co-occur with the original *SLC25A40-ABCB1* fusion [93]. The majority of these fusions were characterized by a fusion of a non-coding exon of the partner gene, fused to exon 2 onwards of *ABCB1*, leaving most of the full gene to be transcribed and translated into a functioning protein [93] (Figure 4A). The presence of *ABCB1* gene fusions correlated with the number of lines of chemotherapy a patient had received, with the more therapy given to a patient, the more likely it was that a fusion was observed [93]. These two studies provide evidence that, at least in ovarian and breast cancer, *ABCB1* gene fusions that retain exon 2 onwards lead to the upregulation of P-gp and are responsible for acquired chemotherapy resistance. This has not yet been established for other cancer types and more research should be conducted to determine whether fusions occur in other malignancies and as a result of different chemotherapy regimens.

### 5.5. SNPs Leading to Changes in ABCB1 Expression

As well as structural variants, single-nucleotide polymorphisms (SNPs) can impact the expression of *ABCB1*. Many of the studies investigating *ABCB1* SNPs are simply correlative, where they establish whether the presence of an SNP leads to better or worse overall survival [94,95,96,97,98]. From studies such as these, it is difficult to discern whether the presence of the SNP is related to changes in *ABCB1* expression or efflux activity, with the possibility that the SNP is completely unrelated to either of those outcomes. Despite this, prior studies have assessed how variants may affect P-gp expression. For example, a study published by Mansoori et al. found that SNPs rs28381943 and rs2032586 (Figure 4B) led to an increase in *ABCB1* mRNA stability in gastric cancer, and therefore increased P-gp expression [99]. Interestingly, both SNPs are intronic, and thought to disrupt splicing, resulting in the retention of intron 19 and 11, respectively [99] (Figure 4B). Moreover, the synonymous SNP *ABCB1* C3435T (Ile1145Ile, isoform 2) (Figure 4B) has been studied extensively and there is currently conflicting evidence as to whether it increases or decreases *ABCB1* mRNA expression. Studies looking at this SNP in the context of intestinal [100], liver [101], and kidney [102] P-gp found expression of *ABCB1* to be lower in individuals harboring the TT genotype compared to the CC genotype. It has been predicted that the C>T substitution changes the secondary structure of the mRNA [101], therefore leading to lower mRNA stability and thus lower levels. Conversely, another study looking at the same SNP (*ABCB1* C3435T) also in the context of intestinal P-gp found that the TT genotype conferred higher P-gp expression compared to the CC genotype [103]. The differences between these findings could stem from the fact that intestinal P-gp was studied in two different populations, with Hoffmeyer et al.’s study being conducted in a Caucasian population [100] and Nakamura et al.’s study being conducted in a Japanese population [103]. This indicates that this association between *ABCB1* C3435T and P-gp expression is more complex than a simple one-to-one correlation. It is also important to note that C3435T is a synonymous SNP, encoding isoleucine regardless of a C or T at position 3435. It is still not fully understood how exactly this single nucleotide change is able to impact the stability of the *ABCB1* mRNA, and more research needs to focus on understanding this in normal tissues before we can move on to trying to understand its role in cancer chemotherapy resistance.

### 5.6. SNPs Leading to Changes in ABCB1 ATPase Activity

*ABCB1* SNPs have also been associated with changes in P-gp efflux activity. While there is much debate on whether *ABCB1* C3435T influences P-gp expression levels, there is a more unified consensus regarding its impact on P-gp efflux activity. A study utilizing leukocytes from 31 healthy donors representing the three genotypes of *ABCB1* C3435T (CC, CT, TT) found that rhodamine 123 efflux was highest in individuals with the CC genotype, followed by the heterozygote CT, and lastly, the TT genotype, which had the lowest levels of rhodamine 123 efflux [104]. Another well-studied synonymous polymorphism is *ABCB1* C1236T (Gly412Gly, isoform 2) (Figure 4B). A study was conducted that aimed to characterize the role of *ABCB1* SNPs on the rate of docetaxel clearance in patients with a variety of solid tumors [2]. Pharmacokinetically, researchers found that patients harboring the homozygous genotype TT experienced a decreased rate of docetaxel clearance [2] (i.e., lower kidney and liver efflux activity in patients with TT compared to those with the CC or CT genotype). Another study conducted in the Caco-2 human adenocarcinoma cell line aimed to determine the impact of the *ABCB1* C1236T polymorphism on the P-gp efflux activity of a number of chemotherapeutic agents [105]. By comparing Caco-2 cell lines stably expressing the wild-type of variant P-gp, they found that cells expressing the wild-type CC genotype transported doxorubicin at a higher rate than variant-expressing cells [105]. Conversely, variant-expressing TT genotype cells transported methotrexate and etoposide more efficiently [105]. Lastly, actinomycin D was found to accumulate at similar levels regardless of whether cells overexpressed the wild-type or variant P-gp [105].

Despite extensive research efforts being focused on these two synonymous variants, there are a few non-synonymous variants that have also been shown to impact P-gp efflux activity. A study found that NIH-3T3 mouse fibroblast cells expressing the mutant *ABCB1* G2677T (TT genotype; Ala893Ser) (Figure 4B) had higher efflux activity of digoxin [106], a drug used to treat heart arrhythmias and a known P-gp substrate. They also conducted a population study among a European American and African American cohort and found the presence of all three SNPs in 62% and 13% of their cohorts, respectively [106]. In subjects with all three of these SNPs, using the levels of P-gp substrate fexofenadine (an antihistamine) as a proxy for transporter activity, this group saw that P-gp efflux activity was higher than those without these SNPs [106]. It is unclear as to whether this haplotype correlates to increased efflux of all P-gp substrates but it is clear that these SNPs are involved in the regulation of P-gp efflux activity, and it is worth investigating their role in cancer cell chemotherapy resistance in the future.

Lastly, a less-well-studied non-synonymous SNP, *ABCB1* G1199A (Ser400Asn, isoform 2) (Figure 4B), has also been found to impact P-gp efflux activity. Results from the rhodamine 123 uptake experiments indicate that LLC-PK1 kidney epithelial cells harboring the AA genotype (Asn-400) have higher efflux activity than wild-type cells (GG, Ser-400) [107]. Additional EC_50_ experiments found that both variant and wild-type cell lines were equally as resistant to doxorubicin but the variant (AA, Asn-400) cells were more resistant to both vinblastine and vincristine [107]. This has important clinical implications for patients in determining their response to treatment.

In the cases of the SNPs C1236T [105], G2677T [106], and G1199A [107], researchers found that *ABCB1* mRNA and P-gp protein expression levels were consistent between the mutant/variant SNP as compared to the wild-type. These results may indicate that these SNPs can enhance/increase P-gp ATPase activity without a significant increase in mRNA or protein expression. As there are few studies in this area, repeating these experiments using multiple different cell lines and in vivo is necessary before such conclusions can be drawn. Furthermore, in all of these cases, the researchers did not investigate the underlying mechanism by which these SNPs were able to enhance P-gp activity; therefore, more research should be performed on this subject in the future.

### 5.7. Epigenetic Mechanisms of ABCB1 Upregulation

**Figure 5:**
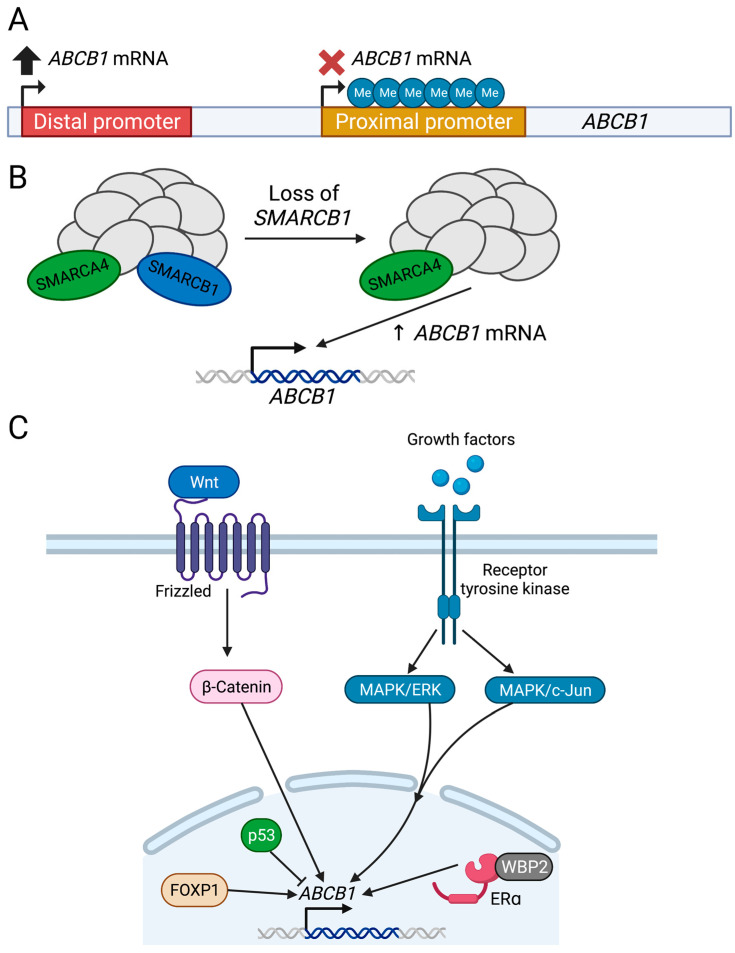
Epigenetic and transcriptional regulation of *ABCB1*: (**A**) Schematic of *ABCB1* gene with proximal and distal promoters. Promoter hypermethylation prevents transcription; (**B**) Regulation of *ABCB1* by chromatin remodeling complex SWI/SNF. *SMARCB1*-deficient SWI/SNF increases *ABCB1* transcription; (**C**) Transcription factors and pathways that activate or inhibit *ABCB1* transcription. Created with BioRender.com (accessed on 4 August 2023).

Epigenetic changes can also lead to the upregulation of *ABCB1*. Here, the *ABCB1* gene can be modified in a way that does not change its underlying sequence or the number of copies of said sequence. One example is promoter methylation (Figure 5A). As outlined in Section 3, it has been established that *ABCB1* has two promoters—an upstream, distal promoter and a downstream, proximal promoter—which are separated by around 110 kb [21]. Transcripts generated from these two promoters will only differ by a few hundred bases, and varying promoter usage is likely the difference between variant 3 and variant 4 mRNAs (as outlined in Table 1), both encoding isoform 2 of P-gp (1280 amino acid isoform). Most cells will utilize the downstream promoter; however, it has been found that multidrug-resistant cells will use the upstream promoter, with a small percentage of *ABCB1* transcripts being derived from this promoter in these cells [21]. Researchers sought to understand why these multi-drug-resistant cells were using this upstream promoter, using the breast cancer cell line MCF7 and its docetaxel-resistant subline [21]. Through the bisulfite sequence, it was found that the downstream promoter of *ABCB1* was hypermethylated in the resistant cells compared to the docetaxel-sensitive cells, thus, forcing the cells to utilize the upstream promoter [21] (Figure 5A). This hypermethylation of the downstream promoter was correlated with *ABCB1* RNA expression, and when resistant cells were treated with 5-aza-2′-deoxycytidine, a DNA hypomethylating agent to promote demethylation of the downstream promoter, *ABCB1* mRNA expression was found to decrease [21]. This downstream promoter hypermethylation has also been observed in the cisplatin-resistant lung adenocarcinoma cell line, A549 [108], and five different taxane-resistant esophageal cancer cell lines [109]. In these studies, the *ABCB1* downstream promoter was hypermethylated also, and demethylation both decreased *ABCB1* RNA expression in the lung [108] and esophageal lines [109], and re-sensitized cells to cisplatin in the case of the lung cancer line, A549 [108]. These three works demonstrate that this mechanism of *ABCB1* upregulation may be common among different cancer types and across different chemotherapeutic agents.

In addition, the SWI/SNF chromatin remodeling complex has also been implicated in *ABCB1* regulation. A forward genetic screen conducted in the near-haploid chronic myeloid leukemia cell line, Hap1, found that upregulation of *ABCB1* and loss of SWI/SNF complex members *SMARCB1* and *SMARCA4* were responsible for doxorubicin resistance [110]. Upon deletion of *SMARCB1* in the Hap1 cells, *ABCB1* RNA was upregulated by 5.8-fold, whereas in *SMARCA4* deleted cells, *ABCB1* RNA was upregulated 1.7-fold [110] (Figure 5B). Corresponding protein studies found that in *SMARCB1*-deleted cells, P-gp was overexpressed but in *SMARCA4*-deleted cells, P-gp was actually downregulated [110]. To untangle how these two members from the same complex could have opposing roles in the regulation of *ABCB1*, researchers engineered a double depleted line (*SMARCB1*^-^ *SMARCA4*^-^) where they saw a downregulation of *ABCB1* RNA as well as protein [110]. To investigate further, they deleted *SMARCB1* in the *SMARCA4*-deficient lung cancer cell line, A549, and saw no change in *ABCB1* RNA levels [110]. Re-expression of *SMARCA4* in the A549 *SMARCB1*-depleted cell line led to an increase in *ABCB1* RNA levels [110] (Figure 5B). This led to the conclusion that *ABCB1* upregulation is dependent on the presence of functional *SMARCA4* and non-functional *SMARCB1* [110]. The exact mechanism by which SWI/SNF regulates *ABCB1* expression has yet to be elucidated, and it is important to note that while there is a correlation, there is no evidence that the SWI/SNF complex directly regulates *ABCB1*—the mechanism of regulation could be indirect; therefore, more research needs to be conducted to determine how this regulation occurs.

### 5.8. Transcriptional Regulation of ABCB1

Alongside genetic and epigenetic regulation of *ABCB1*, studies have sought to understand how the gene is transcriptionally regulated. For example, a study by Choi et al. found that in the ovarian cancer cell lines A2780 and SKOV3, overexpression of the transcription factor *FOXP1* led to an increase in *ABCB1* RNA (Figure 5C), and conferred paclitaxel and cisplatin resistance to those cells [111]. On the other hand, gene silencing of *FOXP1* via shRNA led to a decrease in *ABCB1* RNA and re-sensitized cells to both chemotherapeutic agents [111]. As the study did not establish whether FOXP1 directly transcribes *ABCB1*, it is difficult to conclude that FOXP1 is a direct regulator of *ABCB1*. Regardless, more studies are needed to establish whether this relationship is direct and if it is applicable to other cancers and chemotherapeutic agents. FOXP1 is not the only transcription factor that has been found to regulate *ABCB1* transcriptionally. An early study by Chin et al., utilizing the chloramphenicol acetyltransferase (CAT) reporter gene under the control of the *ABCB1* promoter, found that when mouse fibroblast NIH 3T3 cells expressed a mutant loss-of-function (LOF) *p53* (Arg175His) they had 7-fold higher CAT activity than cells with wild-type *p53* [112]. Co-transfection with wild-type *p53* in these cells found that there was a 50% decrease in CAT activity, indicating that wild-type *p53* might directly or indirectly act as a transcriptional repressor of *ABCB1* [112] (Figure 5C). These results were also validated in the human adrenocortical carcinoma cell line, SW13, which at baseline, had high *ABCB1*-*CAT* fusion RNA expression [112]. The introduction of wild-type *p53* into these cells reduced *ABCB1-CAT* expression by 60-fold, whereas the introduction of the mutant LOF *p53* had no impact on expression [112]. While these in vitro findings suggest *ABCB1* is regulated by *p53*, in vivo studies have not been able to replicate these results. Utilizing 34 colorectal tumors, researchers characterized P-gp expression as well as the presence of *p53* mutations [113]. Here, they found no significant correlation between *p53* mutational presence or protein expression of p53 with P-gp expression, stating that p53-negative (no protein observed) tumors had higher P-gp expression levels compared to p53-positive tumors, although this difference was not significant [113]. From this work, they concluded that p53 does not regulate *ABCB1* in vivo; however, if p53 is a transcriptional repressor of *ABCB1*, and p53-negative tumors had higher P-gp expression, a case could be made that loss of *p53* means loss of *ABCB1* repression and, thus, overexpression of the gene. Regardless, more research should be performed to understand the relationship between *p53* and *ABCB1* both in vitro and in vivo.

There have also been many signaling pathways that have also been implicated in the regulation of *ABCB1*. For instance, the Wnt/β-catenin signaling pathway (Figure 5C). The *ABCB1* basal promoter has several sites for β-catenin binding, prompting researchers to investigate the role of this pathway in *ABCB1* regulation [114]. Using ChIP and vincristine-resistant K562 leukemia cells, it was established that β-catenin was bound to the *ABCB1* promoter at a much higher frequency than in the K562 vincristine-sensitive cells [114]. Using lithium chloride, a Wnt agonist, researchers saw a decrease in phosphorylated GSK3-β as well as increased nuclear translocation of β-catenin, confirming the activation of the pathway [114]. Upon pathway activation, there was an increase in *ABCB1* RNA compared to a vehicle-treated group in both sensitive and resistant cells, indicating that the Wnt/β-catenin pathway can activate the gene [114]. A similar study was also conducted in neuroblastoma cells resistant to doxorubicin [115]. Here, they observed overexpression of the Wnt receptor, Frizzled RNA (*FZD1*), and further analysis saw that this overexpression was correlated to sustained activation of the pathway [115]. *ABCB1* RNA was found to be upregulated in this model, and dual suppression of *FZD1* via shRNA and P-gp inhibition using verapamil saw the re-sensitization of these cells to doxorubicin [115]. Another study in support of this mechanism was conducted in the cholangiocarcinoma cell line, QBC939 [116]. Researchers developed a fluorouracil-resistant subline termed QBC939/5-FU and found that they overexpressed both P-gp and β-catenin compared to their parental counterparts [116]. Upon silencing of β-catenin in these resistant cells, cells became re-sensitized to fluorouracil, which was accompanied by a decrease in P-gp expression. All of these studies together indicate that the Wnt/β-catenin pathway regulates *ABCB1* expression across multiple cancer types and chemotherapies. As these works were conducted in vitro, it would be interesting to see how this translates in vivo, potentially in animal studies, and eventually in patients.

Alongside Wnt, there is evidence that *ABCB1* is also regulated by MAPK pathways (Figure 5C). A study conducted in two acute lymphoblastic leukemia cell lines, CCRF-HSB-2 (T-ALL) and YAMN90 (B-ALL), found that *ABCB1* mRNA increased upon activation of the MAPK/ERK pathway, indicating a potential interaction there [117]; however, it was not established if this regulation is direct or indirect. Similarly, Katayama et al. found that by inhibiting MEK, a component of the MAPK/ERK pathway, P-gp expression in two human colorectal cancer cells, HCT-15 and SW620, was reduced between 5- and 20-fold compared to untreated cells [118]. Once again, it was not established whether this regulation is direct or indirect. As well as the MAPK/ERK, regulation of *ABCB1* via the MAPK/c-Jun pathways has been identified. Using the vincristine-resistant colorectal cancer subline HCT8/V and their parental counterpart, HCT8, researchers found that COX-2 overexpression led to activation of JNK (part of the MAPK/c-Jun pathway), and was correlated with an overexpression of P-gp [119]. By using a JNK inhibitor in combination with a COX-2 inhibitor, *ABCB1* promoter activity decreased, which was accompanied by a drop in *ABCB1* RNA and P-gp [119]. These findings suggest that the MAPK pathways are also able to regulate *ABCB1* expression. Once again, these studies were conducted in vitro, so in vivo studies would need to be performed to determine how applicable this mechanism of regulation is to patients.

Lastly, it has been found that hormone-driven signaling may also regulate *ABCB1* transcription. For example, estrogen signaling. A study conducted by Chen et al. sought to understand the underlying mechanisms of doxorubicin resistance in estrogen receptor alpha (ER-α)-positive breast cancer cells. They found the gene *WBP2* to be overexpressed in the resistant cells compared to both doxorubicin-sensitive cells and ER-α-negative cells [120]. It has been established that WBP2 directly binds and regulates ER-α, leading to the proliferation and promotion of breast cancer [120]. Suppression of this gene re-sensitized the resistant cells to doxorubicin, and upon overexpression of WBP2 in sensitive cells, induced the expression of *ABCB1* [120]. This was specific to the ER-α-positive cells and was not seen in the ER-α-negative cell line [120]. To further elucidate this relationship, ChIP was performed on ER-α-positive, *WBP2*-overexpressing MCF7 cells, where they found the estrogen response element of the *ABCB1* promoter as being co-immunoprecipitated with ER-α [120]. This supports the notion that ER-α is able to positively regulate *ABCB1* in the presence of WBP2 (Figure 5C). Other studies have shown that upon treatment of estrogen, multidrug-resistant ER-α-positive breast cancer cells downregulate P-gp but not *ABCB1* RNA [121,122], suggesting a post-transcriptional mechanism of regulation. In an ovarian cancer model, treatment with estrogen also downregulated P-gp protein without amplification of *ABCB1*; however, they found that treatment with progesterone did increase *ABCB1* RNA, and a combination treatment of estrogen and progesterone led to lower *ABCB1* RNA and P-gp levels [123]. While conflicting results have arisen regarding estrogen- and ER-α-mediated regulation of *ABCB1*, it appears that there is a connection worth studying, and outcomes may have important implications in hormone-driven resistant cancers such as breast and ovarian.

## 6. Conclusions

Although *ABCB1* is one of the most well-studied members of the ABC family of transporters, much remains to be uncovered. As the list of P-gp substrates continues to grow, it remains unclear what exact features dictate whether a compound has the ability to be transported by the efflux pump. Moreover, we need to push forward in our understanding of P-gp isoforms. Different variants of the protein may have different substrates, and, therefore, different roles in normal bodily functions and disease. Lastly, we have a solid foundation for genetic, epigenetic, and transcriptional mechanisms of *ABCB1*/P-gp upregulation in cancer, having identified many of the key players and processes involved; however, many of the proposed pathways have not established whether regulation is direct or indirect, and moving forward we need to characterize exactly how *ABCB1*/P-gp is becoming overexpressed. A deeper understanding will enable us to develop novel therapies to overcome resistance when it occurs or even combination therapies/regimens that circumvent the acquisition of resistance altogether.

## Figures and Tables

**Table 1:** Differences in transcript and protein data between NCBI and Ensembl databases. mRNA length is listed in base pairs and protein length in amino acids.

NCBI Accession Number	Ensembl Accession Number	Number of Exons	mRNA Length (bp)	Protein Length (AA)
NM_001348945.2		32	5586	1350
NM_001348944.2		30	5387	1280
NM_000927.5 NM_001348946.2	ENST00000622132.5	29 28	5534 5205	1280 1280
	ENST00000265724.8	29	4720	1280
	ENST00000543898.5	29	4524	1216
	ENST00000416177.1	6	461	48

**Table 2:** Tissue localization, subcellular localization, and non-cancer drug substrates of human *ABCB1*/P-gp.

Tissue Localization	Subcellular Localization	Substrates
Adrenal gland [24,25] Kidneys [24,25] Colon [24,25] Rectum [24,25] Lungs [24,25] Liver [24,25] Blood–brain endothelial cells [34] Placenta [35]	On the cell surface/within the plasma membrane of: Healthy tissue [25,27,28] Drug-resistant cells [36,37]	Pesticides [38,39] Antibiotics: Puromycin [40] G418 [40] Aureobasidin A [41] Azithromycin [42,43] Amoxicillin [43,44] Clarithromycin [43,44]; Calcium channel blockers [45,46] Protease inhibitors [47,48] Antihistamines [49] Hormones and steroids Cortisol [50,51,52] Aldosterone [50,53,54] Dexamethasone [50,55] Amyloid-β [56] Rhodamine 123 [57]
